# Ununited Fractures

**Published:** 1856-10

**Authors:** W. Parker

**Affiliations:** The President, in the Chair


*Ununited Fractures. Stated Meeting, New York Academy of Medicine, July* 2, 1856. The President, Dr. W. Parker, in the Chair.

The discussion of the subject of Ununited Fractures being in order, it was opened by

Dr. Detmold, who stated the cause of non-union to be both local and constitutional; but sometimes no cause could be assigned. The old method of causing union, was, to excite inflammation between the fractured extremities, by friction, blisters, the seton, &c. Resection and wiring the ends together, was another mode. Dr. D. stated that Dr. Mott's cases (where the seton had been employed) were only successful where it had been necessary to *drill,* in order to introduce the seton; and he thinks that Dr. Mott was misled in this way. Dieffenbach resorted to drilling and fastening with ivory pegs. Five years ago Dr. D. had tried boring with a common gimlet, and was successful in all cases. A Committee from the Academy had seen one of these operations. This method was afterwards claimed by a Western surgeon (Dr. Brainard).

Dr. Buck referred to the local conditions preventing union, such as portions of muscle or tendon getting between the ends of the bone. Dr. B. gave the history of a case in which union of the humerus was prevented in this manner. In the tibia and fibula it is sometimes almost impossible to keep the upper fragment of the tibia in place, and the union of the fibula often kept up this difficulty. Dr. B. spoke of Malgaigne's apparatus for overcoming this displacement. He had both failed and succeeded with both seton and exsection. The perforating method was generally successful in about twelve weeks. Friction was useful where the cases were not of too long standing.

Dr. Detmold stated that he had been successful with the perforating method, where Dr. Buck had failed with the seton. He had also been very successful with a fractured ulna, after the third trial. One great advantage of the perforating method, was, that it leaves no unpleasant symptoms. He had often operated at his office, and allowed the patient to go home. Another advantage was, that it could be repeated any number of times; another, that it does not shorten the limb. He had never failed in the operation. He repeats the operation in one week, if no union has taken place.

Dr. Buck stated that he introduced the seton in a case, by means of perforating, and union followed; but, afterwards, it was again broken, and the patient came under the treatment of Dr. Detmold.

Dr. Parker considered this question important for all practitioners, as it often occurred when no cause could be assigned for it, when there was no fault on the part of the surgeon, and was often the basis of mal-practice suits. In non-union of the lower extremities of hospital patients he used the starch bandage, recommended all possible use of the limb, together with good diet, fresh air, and stimulating drinks. *Excision* (as introduced by White, of Edinburgh, in 1760, and improved by J. K. Rodgers, of this city, by wiring the ends together,) was often successfully employed. *Electricity* is useful; he knew of one case treated successfully by this agent. Dr. P. had noticed that operations on the upper extremities were followed with less success than on the lower, and asked for an explanation of this. He had been successful with the drill; and asked if any benefit was derived from leaving the chips or borings in the hole—he thought there might be.

Dr. Detmold agreed with Dr. Parker that these bony particles, or debris, served as a nucleus for the new union.

Dr. Stone thought that in these cases of fracture it was especially important to ascertain whether the case was one of *delayed union*, or one of *true false-joint*, where the bones were covered with cartilage, and where a new synovial capsule existed. In the upper extremity, particularly in the humerus, false joints were not uncommonly found, and the fractured extremities, if oblique, could not be readily approximated. In such cases he did not believe the operation by the gimlet, as proposed by Dr. Detmold, would be likely to succeed, or that any operation promised success unless it had for its objects the removal of the cartilage and destruction of the capsule, and the approximation of the broken extremities to each other. In the lower extremities, especially in fractures of the tibia, it was extremely rare to meet with cases of false joint. He (Dr. Stone) had never met with a single instance, and was disposed to regard them rather as cases of *delayed union*, which would readily unite if a very little inflammation could be excited. Dr. Stone would expect to obtain this result by the repeated application of blisters; others, by rubbing of the bones together; but, Dr. Detmold, by boring with a gimlet. This suggestion of Dr. Detmold was a useful one; but in the fact of union taking place in a week, it proved that very little inflammation was necessary. Dr. Stone had been on the Committee who investigated Dr. Detmold's cases, and had then come to this conclusion. As to the idea that the dust or debris contributed to the union, he must confess that it seemed to him to be truly fanciful; he should suppose that the dust must necessarily be removed in abstracting the gimlet; or, on the supposition that it might possibly be left behind, he should regard this as an untoward accident, which would be likely to act as a foreign body and produce abscesses.

Dr. R. S. Kissam remarked that he was successful in one case of non-union of the humerus, by means of the starched bandage.

Dr. Batchelder remarked that fractures are solutions of continuity in the bones, which heal in the same way as do like solutions of continuity (wounds) in the soft parts, *i. e.*, by the first or second intention. When the fractured extremities in simple fractures are brought and kept in apposition, the lacerated vessels, pertinent to the bone in the immediate vicinity of the fracture, inosculate, and union by the first intention ensues, as in wounds in the soft parts; with this exception, that the earthy matter which immediately surrounds these vessels at the place of fracture must be first absorbed. This preliminary step is accomplished in a longer or shorter time, according to the age of the patient, or size of the bone. When the crepitus ceases to be perceived, it is an indication that the absorption of the earthy matter is nearly or quite completed; and as motion at the place of fracture diminishes, we infer that re-union is progressing about in proportion to the diminution of abnormal motion; so, when this motion has ceased, the inference is that union has taken place, and that the process of re-deposition of ossific matter among and around the vessels has begun, or will soon begin, and continue till callus is completely formed, provided the fractured parts are kept in position and motionless. If such motion be allowed, the newly-formed vascular union will be broken up, and the re-union will be prevented, and the process have to be gone over with again. When the parts are much disturbed and the union repeatedly broken up, the vessels, thus foiled in their attempts to repair the injury, refuse to renew their efforts, but, uniting with the periosteum, form a ligamentous union, which, while it serves as a connecting medium between the ends of the bone, keeps them asunder, and prevents bony union. To get rid of this interposed ligamentous substance, and cause absorption of the earthy matter surrounding the vessels in the immediate vicinity of the fracture, is the first object which surgery should aim at as essential to the cure. In the larger bones this ligamentous union is very conspicuous, and is most perfect in cases of ununited fracture of the neck of the femur. In these cases, the capsular ligament is folded in between the fractured surfaces, and acts in the first instance as an extraneous body which tends to prevent osseous union, but which in the end contributes mainly to that which is ligamentous. The objects to be aimed at in all our endeavors to cure ununited fracture, are:—1. To get rid of this interposed ligamentous substance; 2. To promote or effect the absorption of the earthy matter which surrounds the vessels at the broken ends of the bone; 3. When this is accomplished, to bring and keep these ends in apposition, that the vessels thus prepared may inosculate and unite as they should do, and probably would have done in the first instance if they had had fair play, or if there had been no fault in the patient's constitution. The method which Dr. B. has adopted and practiced, (with few exceptions in favor of the starch dressing,) is, to wind a bandage firmly around the affected limb, in order to prevent swelling below the part to which the appropriate application is to be made. This done, a piece of thick sole-leather, soaked in water until perfectly soft and pliable, cut and fitted exactly to the limb, and long enough to reach from joint to joint, and sufficiently large to cover the whole, is to be applied and bound firmly on with a bandage. While this dressing is being consolidated, great care must be taken to put and keep the fractured extremities in perfect apposition, or as nearly so as possible; which is best accomplished, when the lower extremity is the affected member, by relays of assistants, who will hold the parts *in situ.* When this dressing has become dry and consolidated, measures should be taken to make the lower fragment press firmly against the upper. As nothing in the system can resist pressure, and as newly-formed parts yield readily to its influence, the interposed ligamentous substance, of which we have spoken, and also the earthy matter immediately surrounding the vessels at the fractured ends, are soon absorbed, and the vessels which have been thus freed of osseous matter will unite, and the union be re-established. In case of a lower extremity, it may be proper to let the patient get up and move about with the help of crutches, and, perhaps, bear more or less weight upon the foot, taking care to do it in such a way as to cause as little motion between the fractured ends as possible; especially after a short time; as motion, if kept up, would infallibly prevent union. If it be the humerus, the patient should be confined to his bed, and an apparatus, besides the rubber-splint, be so contrived and applied as draw the lower fragment up, and force the fractured surfaces to press firmly one against the other, which cannot be so well done in the erect or sitting posture as in the recumbent. This bone (the humerus) is the one in which the artificial joint most frequently occurs, and in which the usual methods of cure have most signally failed. In reflecting upon these facts, it has occurred to Dr. B. that both these accidents were to be imputed to our mode of treating these fractures; splints are firmly bandaged on, which, with the weight of the limb below the fracture, prevent the muscles drawing the lower fragment up, and causing it to make the requisite pressure against the fractured end of the upper portion—in which opinion he has been fully confirmed, when finding at the end of several weeks there was no union: the impending evil has been immediately obviated by applying an apparatus which caused the fractured ends to press one against the other. He believes that this method of treatment will effect a cure in most cases of ununited humeri, even of long standing.

In compound fractures, union takes place by the second intention, which does not usually commence, or is not appreciated, until the external wound is nearly or quite closed. Dr. B. remarked that the starch bandage, the gutta percha splint, and probably the gypsum bandage, as recommended by Dr. Mathysen, (which he had never tried,) will no doubt be found better than the leather splint, which was resorted to many years before anything was known of even the starch bandage, or the "dextrine," as recommended by Prof. Van Buren.

Dr. Batchelder exhibited a specimen from Dr. Finnell's collection, of ununited fracture of the radius and ulna, which beautifully illustrated the manner in which the ligamentous union is formed.

Dr. Finnell remarked, that the man to whom the ulna and radius, referred to by Dr. Batchelder, belonged, had good use of his arm. In St. Vincent's Hospital he had often found it necessary to use stimulants freely. He approved of electricity.

Dr. Phelps observed that all present must conclude, that the main point in practice was to secure a certain amount of inflammation in the ends of the bone before union could be produced.

Dr. W. Parker said, that the question seemed brought to two points:—1. Where there is no union by ligament; and, 2. Where there is a false joint. So long as the inflammation is mild, there is no union. Again, there is such a thing as too much inflammation. Diarrhœa, lactation, burns, suppuration, &c., may prevent union. A physician of this city who had a fracture and an injury of the brain, was severely salivated, and no union took place until the salivation ceased, at the end of thirteen weeks. The system must be brought to the ordinary standing point, by correcting it if deranged, and bringing it to a proper tone. But, in a false joint, an operation must be performed.

Dr. McNulty presented an instrument for the purpose of introducing a platinum wire between the ends of an ununited bone, by means of which he proposed to pass a galvanic current to act as a cautery, and thus produce inflammation and union. Dr. McN. was requested to give the history of the cases in which he might try it.

